# Thyroid-Hormone–Disrupting Chemicals: Evidence for Dose-Dependent Additivity or Synergism

**DOI:** 10.1289/ehp.8195

**Published:** 2005-07-21

**Authors:** Kevin M. Crofton, Elena S. Craft, Joan M. Hedge, Chris Gennings, Jane E. Simmons, Richard A. Carchman, W. Hans Carter, Michael J. DeVito

**Affiliations:** 1 Neurotoxicology and; 3 Experimental Toxicology Divisions, National Health and Environmental Effects Research Laboratory, Office of Research and Development, U.S. Environmental Protection Agency, Research Triangle Park, North Carolina, USA; 2 Solveritas LLC, Richmond, Virginia, USA

**Keywords:** additivity, cumulative risk, polyhalogenated aromatic hydrocarbons, synergism, thyroid hormone disruptors

## Abstract

Endocrine disruption from environmental contaminants has been linked to a broad spectrum of adverse outcomes. One concern about endocrine-disrupting xenobiotics is the potential for additive or synergistic (i.e., greater-than-additive) effects of mixtures. A short-term dosing model to examine the effects of environmental mixtures on thyroid homeostasis has been developed. Prototypic thyroid-disrupting chemicals (TDCs) such as dioxins, polychlorinated biphenyls (PCBs), and poly-brominated diphenyl ethers have been shown to alter thyroid hormone homeostasis in this model primarily by up-regulating hepatic catabolism of thyroid hormones via at least two mechanisms. Our present effort tested the hypothesis that a mixture of TDCs will affect serum total thyroxine (T_4_) concentrations in a dose-additive manner. Young female Long-Evans rats were dosed via gavage with 18 different polyyhalogenated aromatic hydrocarbons [2 dioxins, 4 dibenzofurans, and 12 PCBs, including dioxin-like and non-dioxin-like PCBs] for 4 consecutive days. Serum total T_4_ was measured via radioimmunoassay in samples collected 24 hr after the last dose. Extensive dose–response functions (based on seven to nine doses per chemical) were determined for individual chemicals. A mixture was custom synthesized with the ratio of chemicals based on environmental concentrations. Serial dilutions of this mixture ranged from approximately background levels to 100-fold greater than background human daily intakes. Six serial dilutions of the mixture were tested in the same 4-day assay. Doses of individual chemicals that were associated with a 30% TH decrease from control (ED_30_), as well as predicted mixture outcomes were calculated using a flexible single-chemical-required method applicable to chemicals with differing dose thresholds and maximum-effect asymptotes. The single-chemical data were modeled without and with the mixture data to determine, respectively, the expected mixture response (the additivity model) and the experimentally observed mixture response (the empirical model). A likelihood-ratio test revealed statistically significant departure from dose additivity. There was no deviation from additivity at the lowest doses of the mixture, but there was a greater-than-additive effect at the three highest mixtures doses. At high doses the additivity model underpredicted the empirical effects by 2- to 3-fold. These are the first results to suggest dose-dependent additivity and synergism in TDCs that may act via different mechanisms in a complex mixture. The results imply that cumulative risk approaches be considered when assessing the risk of exposure to chemical mixtures that contain TDCs.

Thyroid-disrupting chemicals (TDCs) are xenobiotics that alter the structure or function of the thyroid gland, alter regulatory enzymes associated with thyroid hormone (TH) homeostasis, or change circulating or tissue concentrations of THs. TDCs include a wide range of chemical structures. Chemicals such as perchlorate inhibit the uptake of iodide into the thyroid gland, with subsequent decrease in iodine-based TH synthesis (Wolff 1998). Other chemicals (e.g., thionamides, amitrole, and ethylenethiourea) decrease TH synthesis by inhibition of thyroid peroxidase (Capen 1997, 1998; Hill et al. 1998; Hurley 1998; McClain 1995). Many classes of xenobiotics alter TH levels by altering catabolic pathways. Polyhalogenated aromatic hydrocarbons (PHAHs) represent one such class of chemicals that induce uridine diphosphoglucuronosyl transferases (UGTs). UGTs glucuronidate THs, and induction of these enzymes increases the elimination of THs (Hill et al. 1998; Hood and Klaassen 2000a; McClain et al. 1989; Oppenheimer et al. 1968).

A major uncertainty regarding the endocrine-disrupting ability of environmental xenobiotics is the potential for additive or synergistic (i.e., greater-than-additive) effects of exposure to mixtures (Daston et al. 2003; International Programme on Chemical Safety 2002). Solving the problem of predicting the effects of chemical mixtures is a daunting task. There are limited studies in the peer-reviewed literature that examine mixtures of TDCs (Desaulniers et al. 2003; Teuschler et al. 2002; Wade et al. 2002). Desaulniers et al. (2003) found that use of 2,3,7,8-tetra-chlorodibenzo-*p*-dioxin (TCDD) toxic equivalents predicted the additive effects of a mixture of coplanar polychlorinated biphenyls (PCBs), polychlorinated dibenzo-*p*-dioxins, and polychlorinated dibenzofurans on circulating thyroxine (T_4_) concentrations in neonatal rats. Wade et al. (2002) subchronically exposed adult male rats to a complex mixture of 16 organochlorines, lead, and cadmium. Effects on thyroid histopathology and hormone concentrations were underpredicted based on assumptions of additivity using published health advisories [e.g., reference doses (RfDs), acceptable daily intakes (ADIs)]. These previous efforts investigated the effects of mixtures without concurrent experimental characterization of the effects of the individual chemicals. This type of approach is useful on a case-by-case basis but does not help answer global issues in the arena of mixtures risk assessments (LeBlanc and Olmstead 2004).

Our present study tested the hypothesis that a mixture of 18 PHAHs acts in a dose-additive manner. The hypothesis was tested using a flexible single-chemical-required (FSCR) method of analysis (Gennings et al. 2004). This model assumes that the effects of the mixture will be predicted by the constraint of Berenbaum’s definition of additivity (Berenbaum 1985). In addition this model allows the calculation of the predicted mixture outcome for chemicals with differing dose thresholds and maximum asymptotes (Gennings et al. 2002, 2004). A short-term oral exposure model (Craft et al. 2002) was used to estimate the impact of 18 PHAHs, both alone and as dilutions of an 18-chemical mixture, on serum T_4_ concentrations. This exposure paradigm allowed for an economic approach to deriving extensive dose–response information (seven to nine doses per chemical) for 18 individual chemicals. Doses associated with a 30% TH decrease from control (ED_30_ estimates) were calculated for each chemical, rather than ED_50_ estimates because some chemicals had asymptotic responses at a 50% decrease. We then tested a mixture of these 18 PHAHs in which the chemical ratios were based on a rough average of concentrations found in breast milk, fish, and other food sources of human exposure (Giesy et al. 1994; Larsen et al. 1994; Patterson et al. 1994; Schecter et al. 1994a, 1994b). Concentrations of the individual chemicals in the undiluted mixture were at least an order of magnitude lower than those found to have significant biologic activity (with the exception of PCB-126, where there was an ~ 16% decrease in T_4_ at the dose found in the highest concentration of the mixture). Last, the exposures ranged from approximately background human body burdens to body burdens similar to some highly exposed populations (DeVito et al. 1995; Liem et al. 2000; Longnecker et al. 2003; Lorber 2002). We used this approach to decrease uncertainty in low-dose extrapolation in mixture testing (Feron and Groten 2002).

## Materials and Methods

### Chemicals.

All individual PHAHs were obtained from Accustandard Corporation (New Haven, CT) or Radian Corporation (Austin TX) at purities > 99%. Non-coplanar PCBs were custom synthesized for 99.9% purity. The following chemicals were tested: 2,3,7,8-tetrachlorodibenzo-*p*-dioxin (TCDD); 1,2,3,7,8-pentachlorodibenzo-*p*-dioxin (PCDD); 2,3,7,8-tetrachlorodibenzofuran (TCDF); 1,2,3,7,8-pentachlorodibenzofuran (1-PCDF); 2,3,4,7,8-pentachlorodibenzofuran (4-PCDF); 1,2,3,4,6,7,8,9-octachlorodibenzo-furan (OCDF); 2,4,4′-trichlorobiphenyl (PCB-28); 2,2′,5,5′-tetrachlorobiphenyl (PCB-52); 3,3′,4,4′-tetrachlorobiphenyl (PCB-77); 2,2′,4,5,5′-pentachlorobiphenyl (PCB-101); 2,3,3′,4,4′-pentachlorobiphenyl (PCB-105); 2,3′,4,4′,5-pentachlorobiphenyl (PCB-118); 3,3′,4,4′,5-pentachlorobiphenyl (PCB-126); 2,2′,3,4,4′,5′-hexachlorobiphenyl (PCB-138); 2,2′,4,4′,5,5′-hexachlorobiphenyl (PCB-153); 2,3,3′,4,4′,5-hexachlorobiphenyl (PCB-156); 3,3′,4,4′,5,5′-hexachlorobiphenyl (PCB-169); and 2,2′,3,4,4′,5,5′-heptachloro-biphenyl (PCB-180). The dose ranges and number of dose groups are provided in Table 1. The PHAH mixture was custom synthesized by Cambridge Isotope Laboratories (Andover, MA) and delivered to the U.S. Environmental Protection Agency (EPA) in corn oil at 3 times the highest tested concentration. Concentrations of individual chemicals in the mixture (Table 2) were verified by chromatographic/mass spectrophotometric analyses (Accustandard Corp., New Haven, CT ). The concentrations used in our present study were determined analytically and varied only slightly from the target concentrations. The ratio of chemicals in the mixture was based on the ratios of PHAHs found in breast milk, fish, and other sources of human exposure (Giesy et al. 1994; Larsen et al. 1994; Patterson et al. 1994; Schecter et al. 1994a, 1994b). All dosing solutions were prepared by dilution of the stock solutions with corn oil (Sigma Chemical Co., St. Louis, MO).

### Animals and dosing.

We obtained female Long Evans rats (23 days of age) from Charles River Laboratory (Raleigh, NC) and allowed them to acclimate for a minimum of 4 days in an animal facility approved by the Association for Accreditation of Laboratory Animal Care before being treated. Two animals were housed per plastic cage (45 cm × 24 cm × 20 cm), with heat-treated pine shaving bedding. They were maintained at 21 ± 2°C with 50 ± 10% humidity on a 12/12-hr light/dark cycle (lights on 0600–1800 hr). Feed (Purina Rodent Chow 5001, Barnes Supply Co., Durham, NC) and tap water were provided ad libitum. All animal procedures were approved by the U.S. EPA Institutional Animal Care and Use Committee.

We dosed rats by oral gavage for 4 consecutive days with each individual chemical to establish dose–effect functions. This paradigm was previously shown to result in dose-related decreases in T_4_ concentrations after exposure to PCB-126 and PCB-153 (Craft et al. 2002). Dose ranges and numbers of dose groups are shown in Table 1. We spaced doses at one-half and one-third log units with the aim to have two to three doses with no measurable response, three to four doses closely spaced around the no-effect level, and two to three doses on the descending portion of the dose response. We used two to three separate blocks of animals (separate groups of animals ordered, dosed, and sampled at different dates) to map the dose response for each chemical. Blocks were used to enable testing of large dose ranges. Numbers of animals per group were not similar for each block and ranged from 4 at some of the highest concentrations to 14 in some low-dose and control groups. Control animals (*n* = 8–14) were dosed with the vehicle (1.0 mL/kg corn oil) only. The mixture study was conducted with serial dilutions of the mixture (*n* = 12/dose). We recorded body weights daily and adjusted dosing volumes daily. Rats were semirandomly assigned to treatment groups by counterbalancing for body weights. On the day following the last dose, animals were randomly sacrificed by decapitation (no anesthesia) between 0800 and 1000 hr. Trunk blood was harvested and allowed to clot on ice for 45–90 min. Serum was obtained by centrifugation of clotted blood at 2,500 rpm at 4°C for 20 min and stored at −80°C until analysis.

### T_4_ assays.

We measured serum total T_4_ by standard radioimmunoassay assay kits (Diagnostic Products Corporation, Los Angeles, CA) and analyzed all samples in duplicate. Intraassay and interassay coefficients of variance for all assays were below 10%. Control group means ranged from 41.7 to 55.6 μg/dL, with an average coefficient of variation of 15.3. All data values were standardized to percentage of control for each chemical [(experimental value/control mean) × 100].

### Statistical modeling.

The definition of additivity (i.e., zero interaction) used is given by Berenbaum (1985) and can be related to the isobologram for a combination of chemicals (Loewe 1953; Loewe and Muischnek 1926) through the interaction index. That is, in a combination of *c* (here, *c* = 18) chemicals, let *E_*i*_*represent the concentration or dose of the *i*th component alone that yields a fixed response (i.e., ED_30_), *y*_0_, and let *x_*i*_*represent the concentration/dose of the *i*th component in combination with the *c* agents that yields the same response. According to this definition, if the substances interact in an additive fashion, then


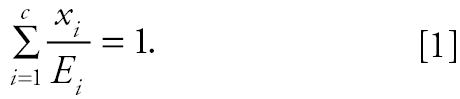


If the left side of Equation 1, termed the interaction index, is < 1, then a greater-than-additive interaction (e.g., synergism) can be claimed at the combination of interest. If the left side of Equation 1 is > 1, then a less-than-additive interaction (e.g., antagonism) can be claimed at the combination.

The 18 chemicals were combined according to a specified mixing ratio (Table 2) and evaluated experimentally. The mixing ratio was selected based on the ratios found in breast milk, fish, and other sources of human exposure, as described above. The mixing ratio is denoted in terms of the proportion, *a_*i*_*, of each chemical in the mixture (Table 2) such that


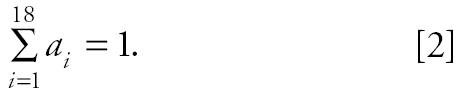


The FSCR approach of Gennings et al. (2004) allows for different threshold parameters and range parameters for each chemical and fixed-ratio mixture. The empirical mixture data were modeled (termed the empirical model) using a nonlinear exponential model of the form


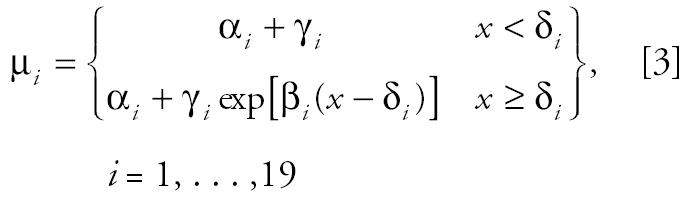


where α*_*i*_*+ γ*_*i*_*= 100, *x* is the dose of the *i*th chemical (*i* = 1, . . . ,18) or the mixture dose for the mixture ray (*i* = 19), α*_*i*_*is an unknown parameter defined by the maximum effect for the *i* th chemical or mixture, β*_*i*_*is an unknown parameter defined by the slope for the *i* th chemical or mixture, and δ*_*i*_*is an unknown parameter defined by the threshold along the *i* th ray for single chemicals (in terms of dose) or mixtures (in terms of total dose of the mixture). Preliminary inspection of the data provided evidence that the variance of the observed response increased with the mean [i.e., Var(*Y* ) = τμ for unknown dispersion parameter τ]. Unknown parameters were estimated using the method of maximum quasi likelihood (McCullagh and Nelder 1989). The model in Equation 3 was used to estimate the ED_30_ (i.e., the dose associated with a 30% decrease from control) for each single chemical. The delta method (Agresti 2002) was used to estimate large sample 95% confidence intervals on the ED_30_ estimates.

**Figure 1:**
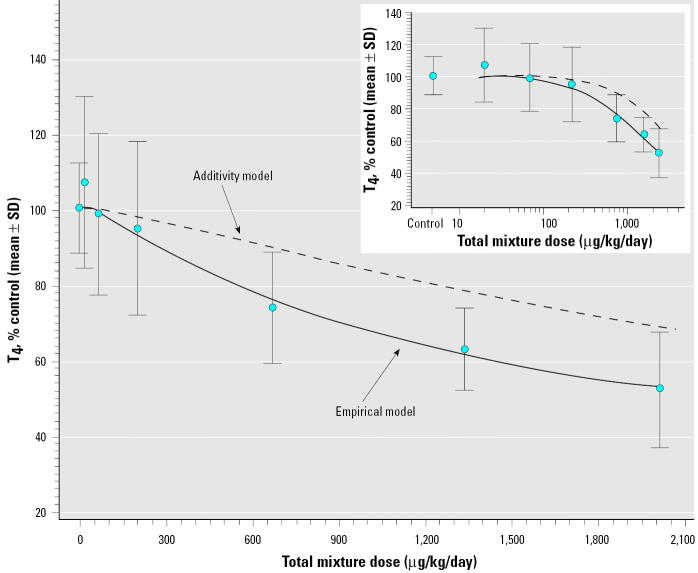
Predicted effects on serum total T_4_ from the single-chemical data in an additivity model and empirical effects of the PHAH mixture. Results demonstrate a significant deviation from additivity at the three highest mixture doses. The effects of the lower mixture doses were not significantly different than that predicted by additivity. The inset illustrates the same data plotted as log-dose.

We estimated the mixture dose–response curve (Figure 1, empirical model) using the model in Equation 3 for the specified mixture. The dose–response curve for the mixture assuming additivity (Figure 1, additivity model) was estimated using only the single-chemical dose–response model parameters and the single-chemical data (Equation 3), then predicting along the mixture ray with the constraint of additivity given in Equation 1.

To determine whether there was a statistically significant deviation from additivity, we used a quasi-likelihood ratio test to compare the empirical mixture model to the restricted additivity model based on an *F*-distribution. The restricted additivity model (Gennings et al. 2004) included only the single-chemical dose–response model parameters but used both the single-chemical and mixture data. We used this restricted model to predict the mean responses for the mixture data using the constraint of additivity given in Equation 1. In addition we compared the predicted responses from the mixture data under the hypothesis of additivity (following the methods in Gennings et al. 2002) to the observed sample means using an *F*-test (df = 6, 1,305).

## Results

We noted no visible signs of toxicity after the short-term PHAH treatments. There were no treatment-related effects on body weight gain. Summary statistics for the single-chemical curve fits, including ED_30_ estimates and 95% confidence limits, are shown in Table 3. Summary statistics for the individual chemical dose groups can be found in Supplemental Data Table 1 (http://ehp.niehs.nih.gov/docs/2005/8195/supplemental.pdf).

Data modeling with the FSCR method provided maximum effect parameters (asymptotes) and dose threshold parameters for each chemical and for the mixture. Note that the dose–response model for OCDF was reduced to background (100%) because the slope parameter (β) was not significant (*p* = 0.84) and the maximum effect parameter was not different from 100%. The estimates for the maximum effect parameters (α) for the single chemicals clustered into three groups, with maximums at 14, 31, and 50% of control (Table 4). The data were also modeled with a single-chemical-required (SCR) approach that requires similar asymptotes (Casey et al. 2004). This model proved to be inadequate with significant lack of fit because of clearly different asymptotes (*p* < 0.0001). The FSCR model proved more appropriate as it allowed for three different asymptotic levels, dose thresholds, and no overall lack of fit (*p* > 0.05).

Table 5 presents summary statistics for the mixture data. The data reveal a mixture-dose–dependent decrease in T_4_ concentrations that produced a maximal decrease of approximately 50%. The experimental mixture data and the fits of the empirical and additivity models to the mixture data are shown in Figure 1. Comparison of the dose–response curve for the mixture under the hypothesis of additivity (Figure 1, dashed line) to the fit to the empirical data (Figure 1, solid line) illustrate the dose-dependent nature of the nonadditive effects of the mixture (Figure 1). A quasi-likelihood ratio test rejected the hypothesis of additivity (*p* < 0.001). Table 6 lists the results of an overall (6 df) test of additivity where the null hypothesis is that the true mean is equivalent to the predicted mean from the FSCR model. From this test, there is statistically significant evidence of departure from additivity (*p* < 0.001). Associated with this test are the six comparisons at each mixture-dose group (Table 6). The T_4_ mean for the mixtures at the three highest mixture-dose groups (667, 1,335, and 2,002 μg/kg/day) were each significantly different from that predicted under additivity. Because the sample means are below that predicted under additivity, there is evidence of an interaction (greater than additivity) at these dose groups. The difference between the additivity model and empirical data (Figure 1) at the three highest mixture doses (i.e., area of maximal difference) was approximately 15% in terms of T_4_ concentration or 2.5-fold on a microgram per kilogram per day dose basis. There was no evidence for significant departure from additivity at the three lowest doses of the mixture (Table 6).

## Discussion

The present study tested the hypothesis that a mixture of TDCs affect T_4_ concentrations in a dose-additive manner. We designed the mixture so that highest mixture-dose levels of the individual chemicals were at or below their no observed effect levels. The FSCR additivity model analyses demonstrate cumulative effects of low doses of the mixture and synergistic cumulative effects of the highest dosages of the mixture. These data advocate consideration of cumulative risk approaches when assessing the risk of exposures to chemical mixtures that contain TDCs.

The single-chemical and mixture data were modeled successfully using the FSCR model. Results demonstrate a very wide range of effective doses of PHAHs that decrease TH concentrations. These findings confirm previous work demonstrating that short-term exposure to TCDD (Craft et al. 2002), and some individual PCB congeners for example, PCB congeners 47, 95, 101, and 153 (Craft et al. 2002; Khan et al. 2002; Saeed and Hansen 1997) cause hypothyroxinemia in the rat. Our present work expands these findings by providing dose–response data and relative potencies for 2 dioxins, 4 furans, and 12 PCB congeners. OCDF was not effective at the doses used in this animal dose model. This was expected because of the limited absorption of this fully chlorinated dibenzofuran (Birnbaum and Couture 1988; DeVito et al. 1998). In addition the ED_30_ estimates provide a basis for establishing relative potency values for these chemicals.

Analyses of the mixtures data demonstrated a dose-dependent synergy. The additivity model underestimated the actual toxic effect of the mixture at the three highest doses tested (Figure 1). Effects of the three lowest doses of the mixture were not significantly different than that predicted by the additivity model. These conclusions are based on the use of the FSCR method (Gennings et al. 2004). These data were also analyzed using an SCR method (Gennings et al. 2002). Although the SCR model provided significant evidence of a greater-than-additive effect (data not shown), this model was not appropriate for use with these data because of significant lack of fit to the data. The SCR model assumes a similar asymptote for all single chemicals and the mixture, a condition not satisfied in the present data set. Use of the FSCR model allowed for multiple asymptotic levels and dose thresholds and resulted in a model with no overall lack of fit.

Three conclusions are apparent from these data. The first is that exposure to the 18 chemical mixture results in dose-dependent greater-than-additive effects on T_4_ concentrations at the highest mixture doses. This conclusion is supported by the FSCR analysis. The second conclusion is that although the greater-than-additive effects are statistically significant, the magnitude of underestimation of the experimental data (Figure 1) by the additivity model (Figure 1) is not large. On a dose basis, the underestimation is about 2.5-fold for the three highest doses of the mixture (Figure 1). This suggests that, even in the high mixture-dose region, the effects of this mixture are predicted by additivity with a fair degree of accuracy. The third conclusion is that departure from additivity was not detected in the low-dose region. Although this suggests that dose additivity predicts effects on T_4_ at low exposures, it is tempered by a presumed low statistical power to detect differences in this area of the dose response.

A significant finding in the present experiment is that the mixture actually caused decreases in T_4_ concentrations. This occurred even though the individual chemical concentrations in the mixture were below effective doses. For example, at the second highest mixture dose there was a 38% decrease in T_4_. The individual dose of PCB-153 at this mixture dose was approximately 254 μg/kg/day. The lowest effective dose of PCB-153 administered alone is much greater than 2,000 μg/kg/day. This relationship was similar for all the chemicals in the mixture with one exception, PCB-126. The dose of PCB-126 in the highest dose of the mixture caused about a 16% decrease in T_4_. These data clearly demonstrate the principle that simple mathematical addition of effects (i.e., effect addition) of individual chemicals will not predict the effects of these TDCs in a mixture.

The biologic reasons for the greater-than-additive effect of this mixture are currently unknown. Risk assessment approaches to additivity assume, where data are lacking otherwise, that chemicals with similar modes of action act in a dose-additive fashion (U.S. EPA 1986, 2000). Although all the chemicals used here decrease circulating T_4_ concentrations, they may do so via a number of different mechanisms. One postulated mechanism for the reduction in T_4_ concentrations is the up-regulation of hepatic UGT isoforms that glucuronidate T_4_, leading to biliary elimination (Capen 1997; DeVito et al. 1999; Hill et al. 1998; McClain et al. 1989). Evidence suggests that UGT1A1 and UGT1A6 are responsible for T_4_ glucuronidation in the rat (Vansell and Klaassen 2002; Visser et al. 1993). These UGT isoforms are induced by aryl hydrocarbon receptor (AhR), constitutive androstane receptor (CAR), and pregnane-X receptor (PXR) agonists. The dioxins, furans, and coplanar PCBs (e.g., PCB-77, PCB-126) all activate AhR (Wilson and Safe 1998), whereas the more non-coplanar PCBs (e.g., PCB-52, PCB-138, PCB-153) act via CAR/PXR pathways (Connor et al. 1995; Tabb et al. 2004). Some of the chemicals tested (e.g., PCB-105, PCB-118) are agonists for AhR, PXR, and CAR. Activation of these UGTs through the different nuclear receptors may play a role in the synergistic effects. Differential regulation of microsomal enzymes that glucuronidate T_4_ versus T_3_ (triiodothyronine) may also be responsible (Hood and Klaassen 2000a). There are a number of other postulated mechanisms for altering circulating and tissue levels of THs. Hydroxylated metabolites of PCBs displace T_4_ from transthyretin, a major serum transport protein in rats (Brouwer et al. 1998). This mechanism has been hypothesized to decrease bound T_4_, resulting in greater uptake, catabolism, and elimination of T_4_ (Van den Berg et al. 1991). PCBs also alter deiodinases and therefore iodination of THs (Hood and Klaassen 2000b; Morse et al. 1993). There is some evidence that PCBs increase uptake of T_4_ into the liver (Martin 2002), possibly by altering thyroid transporters (Guo et al. 2002). In addition, Khan and Hansen (2003) and colleagues have demonstrated decreased pituitary sensitivity to thyroid-stimulating hormone by two PCB congeners. Therefore, the synergistic effect may be the result of activation of multiple pathways by the mixture, with the measured effect, T_4_, a common downstream end point for these pathways.

The curve fits to the individual chemical data revealed three levels of maximum efficacy (Table 4). Because of the limited number of chemicals, it is difficult to quantitatively describe the structure–activity relationship for maximal T_4_ decreases. In addition the dose–response determinations were not designed to allow prediction of the asymptotic efficacy but instead aimed to characterize the low end of the dose–response functions. In some cases the maximal efficacy was driven by the highest dose tested, which did not demonstrate a clear maximal effect (e.g., PCB-28, PCB-52, PCB-169). The data do support, with a number of exceptions, a rough separation of chemicals into the more dioxin-like chemicals at the 50% point, and mono- and di-*ortho* substituted chemicals having an asymptote at 14%. A likely explanation for the different efficacies is that the PHAHs act through a variety a mechanisms, as discussed above, and the interaction of these mechanisms differentially affects T_4_ levels.

The significance of these findings for environmental exposures is tempered by some uncertainty. In our present study we used a weanling animal model with a short (i.e., 4-day) exposure duration. Short exposure durations, coupled with differences in half-lives of the chemicals in the mixture that vary from a few weeks to many months (Van den Berg et al. 1994), yield potential pharmacokinetic differences that may confound extrapolation of these results. Pharmacokinetic differences between short-term and steady-state exposures may also include differences in saturation of induction and metabolite generation. Thus, extrapolation of our present findings to chronic exposures should be moderated by these uncertainties.

Extrapolation of our present work in rats to humans is tempered by the uncertainty in how the mode(s) of action of the TDCs may differ between species. Current hypotheses on the mechanisms by which PHAHs decrease T_4_ include up-regulation of hepatic UGTs and sulfotransferases, direct effects on the thyroid gland, and displacement of T_4_ from serum transport proteins (Brouwer et al. 1998). Cross-species extrapolation of these mechanisms is difficult (Crofton 2004). In addition one must consider the degree of TH disruption that will lead to adverse outcomes. Small decreases (~25%) in maternal T_4_ during the early fetal period will lead to adverse neurofunctional outcomes (i.e., IQ scores) in humans (Haddow et al. 2002; Morreale de Escobar et al. 2000). Limited data in animals suggest that T_4_ decreases need to exceed 50% before adverse outcomes can be detected (Crofton 2004).

A limited number of studies have examined the effects of complex mixtures of endocrine-disrupting chemicals (EDCs) (Desaulniers et al. 2003; Tinwell and Ashby 2004; Wade et al. 2002). Desaulniers et al. (2003) examined the effects of a mixture of 16 coplanar PCBs, PCDDs, and PCDFs on T_4_ concentrations in neonatal rats. Decreases in T_4_ were associated with dioxin equivalents using the toxic equivalency factor methodology (Desaulniers et al. 2003; Van den Berg et al. 1998). Consistent with our present findings, Wade et al. (2002) found that effects on thyroid histopathology and hormones were underpredicted based on additivity of published health advisories (e.g., RfDs and ADIs). Evaluation of different models for determining the effects of a mixture of seven EDCs on uterotrophic responses led to a conclusion that the most expedient method is to bioassay the mixture rather than test individual chemicals (LeBlanc and Olmstead 2004; Tinwell and Ashby 2004). These studies lack, either by study design or statistical approach, the ability to test for additivity. The present work expands the previous work by applying a rigorous statistical analysis to test for additivity.

## Conclusions

The present work demonstrates that the cumulative effect of a mixture of TDCs is predicted by additivity at low doses and synergy at high doses. These data suggest that low doses of heterogeneous TDCs that alter thyroid homeostasis should be considered together when calculating the risk of exposures to mixtures. Future work should endeavor to expand these conclusions to low-dose chronic exposures and broaden testing of mixtures to include chemicals from diverse classes of thyroid disruptors such as TH synthesis inhibitors.

## Supplementary Material

Supplemental Data Table 1

## Figures and Tables

**Table 1:** Chemicals tested, dose ranges, and number of doses, for individual chemicals.

Chemical	Dose range (μg/kg/day)	No. of doses^a^
TCDD	0.0001–10	10
PCDD	0.003–10	10
TCDF	0.3–100	7
1-PCDF	0.03–100	7
4-PCDF	0.03–90	9
OCDF	0.1–300	8
PCB-28	100–90,000	9
PCB-52	100–90,000	9
PCB-77	100–30,000	8
PCB-101	50–30,000	9
PCB-105	90–90,000	8
PCB-118	10–10,000	9
PCB-126	0.001–100	10
PCB-138	100–90,000	9
PCB-153	100–90,000	9
PCB-156	10–10,000	8
PCB-169	1–1,000	8
PCB-180	100–90,000	8

a Includes a control group.

**Table 2:** Chemical composition of the mixture.

Chemical	Concentration^a^ (μg/mL)	Ratio (TCDD)	Ratio (total mass)
TCDD	0.013	1.0	0.000007
PCDD	0.013	1.0	0.000007
TCDF	0.019	1.4	0.000010
1-PCDF	0.006	0.4	0.000003
4-PCDF	0.026	1.9	0.000013
OCDF	0.065	4.6	0.000032
PCB-28	78.600	5,605.3	0.039237
PCB-52	155.200	11,074.7	0.077523
PCB-77	2.000	141.1	0.000988
PCB-101	153.800	10,973.4	0.076814
PCB-105	76.700	5,468.9	0.038282
PCB-118	381.100	27,186.0	0.190302
PCB-126	0.610	43.1	0.000302
PCB-138	380.900	27,168.7	0.190181
PCB-153	382.200	27,265.9	0.190861
PCB-156	13.100	934.4	0.006541
PCB-169	0.400	28.1	0.000197
PCB-180	377.900	26,957.1	0.188700

a Chemical concentration in the highest dose of the mixture administered.

**Table 3:** Summary statistics for individual chemicals, and the ED_30_ and 95% confidence intervals for each chemical.

Chemical	ED_30_ (μg/kg/day)	95% Confidence interval	αAsymptote estimate^a^
TCDD	0.15	(0.08, 0.22)	50
PCDD	1.51	(1.10, 1.92)	31
TCDF	4.65	(1.90, 7.40)	50
1-PCDF	15.6	(10.17, 21.01)	50
4-PCDF	27.5	(17.05, 38.01)	50
OCDF	—	—	—
PCB-28	76,103.0	(50,142, 102,064)	50
PCB-52	33,025.0	(20,958, 45,092)	50
PCB-77	852.0	(655, 1,049)	31
PCB-101	4,833.0	(3,819, 5,847)	31
PCB-105	1,031.0	(861, 1,200)	14
PCB-118	1,289.0	(1,103, 1,475)	14
PCB-126	1.33	(0.77, 1.88)	50
PCB-138	8,001.0	(6,692, 9,310)	14
PCB-153	12,696.0	(10,659, 14,732)	14
PCB-156	760.0	(629, 891)	14
PCB-169	227.0	(167, 286)	31
PCB-180	30,541.0	(23,122, 37,960)	31

a Percentage of control (e.g., α= 14 represents an 86% decrease in T_4_ concentration relative to the control mean).

**Table 4:** Parameter estimates from the FSCR model.

Parameter	Estimate	SE	*p*-Value
α_1,2,6,12,13,16,17_	50.26	1.45	< 0.001
α_3,10,11,14,15_	30.74	1.95	< 0.001
α_4,5,7,8,9_	14.33	1.03	< 0.001
α_mix_	42.29	14.78	0.004
β_1_ (1-PCDF)	−0.0608	0.0164	< 0.001
β_2_ (4-PCDF)	−0.0378	0.0142	0.008
β_3_ (PCB-101)	−0.000119	0.000020	< 0.001
β_4_ (PCB-105)	−0.000833	0.000171	< 0.001
β_5_ (PCB-118)	−0.000696	0.000109	< 0.001
β_6_ (PCB-126)	−0.719000	0.225	0.001
β_7_ (PCB-138)	−0.000054	0.000006	< 0.001
β_8_ (PCB-153)	−0.000034	0.000003	< 0.001
β_9_ (PCB-156)	−0.000567	0.000061	< 0.001
β_10_ (PCB-169)	−0.002616	0.000549	< 0.001
β_11_ (PCB-180)	−0.000021	0.000005	< 0.001
β_12_ (PCB-28)	−0.000012	0.000003	< 0.001
β_13_ (PCB-52)	−0.000028	0.000006	< 0.001
β_14_ (PCB-77)	−0.000666	0.000102	< 0.001
β_15_ (PCDD)	−0.374900	0.0639	< 0.001
β_16_ (TCDD)	−6.505000	2.414	0.007
β_17_ (TCDF)	−0.331000	0.274	0.228
θ_mix_	−0.000872	0.000467	0.062
δ_1_	0.389	1.500	0.795
δ_2_	3.094	3.927	0.431
δ_3_	76.410	398.449	0.848
δ_4_	513.1	147.9	0.001
δ_5_	669.5	147.5	< 0.001
δ_6_	0.043	0.130	0.742
δ_10_	9.812	25.104	0.696
δ_11_	3,227.3	5,581.7	0.563
δ_16_	0.004	0.020	0.855
δ_17_	1.859	0.977	0.057
δ_mix_	49.5	74.3	0.506

**Table 5:** Effects of PHAH mixture on serum T_4_ concentrations.

Mixture dose (μg/kg/day)	Test solution (% stock)	T_4_ mean (% control ± SD)	Sample size
0	—	100.0 ± 11.8	12
20.0	0.33	106.8 ± 22.7	12
66.7	1.1	98.5 ± 21.7	12
200.3	3.3	94.6 ± 22.9	12
667.5	11	73.7 ± 14.6	12
1,335.1	22	62.9 ± 10.8	12
2,002.6	33	52.2 ± 15.1	12

**Table 6:** Test results for the hypothesis that mean T_4_ values for the mixture dose are equal to those predicted under the additivity model.

Statistical test	Mixture dose (μg/kg/day)	Statistic	*p*-Value
Overall *F*-test (df 6, 1,305)	—	3.43	0.002
Individual *F*-tests (df 1, 1,305)	20.0	1.65	0.200
	66.7	0.03	0.862
	200.3	0.21	0.647
	667.5	8.76	0.003*
	1,335.1	7.91	0.005*
	2,002.6	10.10	0.002*

*Dose groups where the mean T_4_ response is significantly different (*p* > 0.05) from that predicted under additivity.
