# Supplementary material for: Thyroid-Hormone–Disrupting Chemicals: Evidence for Dose-Dependent Additivity or Synergism
Source: Environ Health Perspect. 2005 Jul 21;113(11):1549–54. doi: 10.1289/ehp.8195 (PMC1310917; doi:10.1289/ehp.8195)
Supplement: Supplemental Data Table 1 [file ehp0113-001549s1.pdf]

EHP Manuscript #8195
Title: Thyroid Hormone Disrupting Chemicals: Evidence for Dose-Dependent Additivity or Synergism
Authors: Crofton et al.

This data is for informational purposes only.

Any one wishing to use this data should request permission from:
Kevin M. Crofton, Ph.D.
Neurotoxicology Division, MD-B105-04
National Health and Environmental Effects Research Laboratory
US Environmental Protection Agency
Research Triangle Park, NC 27711 USA
phone: 1-919-541-2672
email: crofton.kevin@epa.gov

Supplemental Data Table 1: Chemicals tested, doses (ug/kg/day), group mean serum total thyroxine (T4) concentrations expressed as % of control, standard deviations and group sample sizes.

Chemical	Dose (µg/kg)	T4 (%control) Mean	T4 (%control) Standard Deviation	Sample Size
1-PCDF	0	99.999	15.652	11
1-PCDF	0.3	101.015	19.888	12
1-PCDF	1	102.128	24.041	11
1-PCDF	3	88.819	15.438	12
1-PCDF	10	77.569	15.569	11
1-PCDF	30	59.427	10.981	12
1-PCDF	100	58.762	20.718	11
4-PCDF	0	100.000	13.818	14
4-PCDF	0.03	111.194	21.411	8
4-PCDF	0.09	101.315	12.244	8
4-PCDF	0.3	105.139	16.756	8
4-PCDF	0.9	115.543	22.292	8
4-PCDF	3	110.321	17.563	8
4-PCDF	9	82.519	19.648	14
4-PCDF	30	72.889	17.945	8
4-PCDF	90	50.393	14.416	14
PCB101	0	100.000	18.986	8
PCB101	50	102.393	12.566	4
PCB101	100	114.366	29.794	10

Chemical	Dose (μg/kg)	T4 (%control) Mean	T4 (%control) Standard Deviation	Sample Size
PCB101	300	86.313	17.605	10
PCB101	1000	87.985	14.636	10
PCB101	3000	80.831	19.946	8
PCB101	10000	52.214	10.388	8
PCB101	20000	39.363	10.256	8
PCB101	30000	30.429	6.124	10
PCB105	0	100.001	12.092	8
PCB105	90	114.703	22.407	8
PCB105	300	93.803	6.664	8
PCB105	900	76.420	15.748	8
PCB105	3000	25.053	12.353	8
PCB105	9000	16.269	7.043	8
PCB105	30000	11.564	0.784	8
PCB105	90000	11.255	0.938	8
PCB118	0	100.413	9.867	15
PCB118	10	81.331	22.058	8
PCB118	30	103.804	13.107	11
PCB118	100	98.513	22.805	8
PCB118	300	93.559	14.718	8
PCB118	1000	81.874	23.969	8
PCB118	3000	32.838	11.139	8
PCB118	5600	13.578	4.088	6
PCB118	10000	16.740	6.638	8
PCB126	0	99.514	12.204	14
PCB126	0.001	99.560	25.634	6
PCB126	0.03	97.754	13.673	14
PCB126	0.1	98.886	13.878	14
PCB126	0.3	89.938	24.415	8
PCB126	1	74.951	11.776	8
PCB126	3	57.039	12.037	8
PCB126	10	51.751	15.813	8
PCB126	30	41.590	12.499	8
PCB126	100	49.341	7.851	8
PCB138	0	99.999	16.362	8
PCB138	100	90.965	3.227	4
PCB138	300	95.553	18.368	8
PCB138	1000	84.041	14.638	8
PCB138	3000	89.485	11.964	8
PCB138	5600	73.453	12.668	4
PCB138	10000	50.553	8.634	8
PCB138	30000	38.765	11.815	8

Chemical	Dose (μg/kg)	T4 (%control) Mean	T4 (%control) Standard Deviation	Sample Size
PCB138	90000	15.613	8.732	8
PCB153	0	100.001	13.091	12
PCB153	100	98.310	5.527	4
PCB153	300	96.795	17.148	4
PCB153	900	99.865	8.651	6
PCB153	3000	88.922	19.104	5
PCB153	9000	75.028	7.644	6
PCB153	20000	53.435	12.969	6
PCB153	30000	46.197	6.119	6
PCB153	90000	20.723	6.230	6
PCB156	0	100.000	11.147	8
PCB156	10	86.759	7.638	8
PCB156	30	92.525	15.392	8
PCB156	100	88.823	16.421	8
PCB156	300	74.916	17.757	8
PCB156	1000	60.943	11.559	8
PCB156	3000	33.724	9.367	8
PCB156	10000	13.030	4.949	4
PCB169	0	100.000	13.325	8
PCB169	1	106.939	25.448	8
PCB169	3	104.790	16.615	8
PCB169	10	103.708	19.038	8
PCB169	30	95.630	19.626	7
PCB169	100	81.838	14.800	8
PCB169	300	63.114	11.918	8
PCB169	1000	37.710	16.273	8
PCB180	0	100.000	13.455	8
PCB180	100	103.326	17.035	8
PCB180	300	110.624	12.279	8
PCB180	1000	94.574	15.458	8
PCB180	3000	109.364	17.082	8
PCB180	10000	93.634	16.874	8
PCB180	30000	66.164	8.490	8
PCB180	90000	44.545	18.197	8
PCB28	0	100.000	5.150	8
PCB28	100	103.668	9.057	4
PCB28	300	101.840	14.480	8
PCB28	1000	91.534	8.957	8
PCB28	3000	103.394	9.759	8
PCB28	10000	91.667	25.897	9

Chemical	Dose (μg/kg)	T4 (%control) Mean	T4 (%control) Standard Deviation	Sample Size
PCB28	20000	86.305	5.111	8
PCB28	30000	79.760	20.061	8
PCB28	90000	69.500	15.826	9
PCB52	0	100.003	15.101	8
PCB52	100	106.565	11.514	4
PCB52	300	99.211	12.965	8
PCB52	1000	95.510	13.985	8
PCB52	3000	93.687	21.082	10
PCB52	10000	84.838	16.019	10
PCB52	20000	77.041	10.601	8
PCB52	30000	75.064	16.654	8
PCB52	90000	54.551	5.611	8
PCB77	0	100.001	11.925	16
PCB77	10	99.578	27.509	6
PCB77	100	88.723	21.404	10
PCB77	300	88.118	27.315	12
PCB77	1000	56.761	27.283	8
PCB77	3000	47.649	8.005	8
PCB77	10000	30.890	13.782	8
PCB77	30000	26.394	15.541	8
PCDD	0	100.000	13.828	7
PCDD	0.003	101.165	8.379	4
PCDD	0.01	83.275	19.123	8
PCDD	0.03	89.815	19.692	8
PCDD	0.1	81.388	20.330	8
PCDD	0.3	83.143	30.634	8
PCDD	1	66.505	14.203	8
PCDD	3	61.728	21.672	8
PCDD	10	33.078	6.188	4
TCDD	0	99.999	15.437	14
TCDD	0.0001	96.267	14.983	6
TCDD	0.003	98.573	18.111	12
TCDD	0.01	99.758	19.039	6
TCDD	0.03	93.323	12.109	6
TCDD	0.1	70.940	12.741	6
TCDD	0.3	62.517	14.754	6
TCDD	1	52.680	22.730	6
TCDD	3	54.663	19.708	6
TCDD	10	49.145	11.146	4
TCDF	0	100.001	15.412	8
TCDF	0.3	94.380	19.822	8

Chemical	Dose (μg/kg)	T4 (%control) Mean	T4 (%control) Standard Deviation	Sample Size
TCDF	1	74.490	18.839	8
TCDF	3	83.520	16.257	8
TCDF	10	50.976	15.189	8
TCDF	30	48.144	9.385	8
TCDF	100	44.991	9.530	8
OCDF	0	100.000	7.524	8
OCDF	0.1	98.914	12.099	8
OCDF	0.3	106.764	8.866	8
OCDF	1	102.525	12.158	8
OCDF	3	110.031	12.939	8
OCDF	30	98.754	8.298	8
OCDF	300	95.161	10.314	8
